# Interplay between *CDH1* polymorphisms, haplotypes, and genomic repetitive elements in urothelial bladder cancer prognosis

**DOI:** 10.1007/s11033-026-12162-6

**Published:** 2026-06-23

**Authors:** Laís Capelasso Lucas Pinheiro, Maria Alice Feitosa de Souza Martins, Maria Fernanda Vicente Turim, Isabely Mayara da Silva, Janaina Nicolau de Oliveira, Fernando Terziotti, Juliana Mara Serpeloni, Karen Brajão de Oliveira, André Luís Laforga Vanzela, Roberta Losi Guembarovski

**Affiliations:** 1 Laboratory of Mutagenesis and Oncogenetics, Department of General Biology, Londrina State University, Londrina, PR Brazil; 2 Laboratory of Molecular Genetics and Immunology, Department of Pathological Sciences, Londrina State University, Londrina, PR Brazil; 3 Cancer Hospital of Londrina – HCL, Londrina, PR Brazil; 4 Laboratory of Cytogenetics and Plant Diversity, Department of General Biology, Londrina State University, Londrina, PR Brazil

**Keywords:** E-cadherin, rs16260, rs7186053, Tumoral recurrence, SINEs

## Abstract

**Background:**

Urothelial Bladder Cancer (UBC) remains a significant clinical challenge due to its high morbidity, substantial economic burden, and high recurrence rates. E-cadherin, an important regulator of cell-cell adhesion encoded by the *CDH1* gene, is frequently downregulated during malignant progression, serving as a hallmark of invasiveness. This study explored the prognostic significance of *CDH1* genetic variants (rs16260 C > A and rs7186053 A > G) and haplotypes, while integrating bioinformatic profiling of repetitive elements.

**Methods and results:**

A cohort of 334 UBC patients was genotyped via qPCR, followed by haplotype reconstruction and association with clinical outcomes. This analysis was complemented by immunofluorescence to illustrate the interplay between tissue invasion levels and *CDH1* genetic backgrounds in selected samples. Bioinformatic tools characterized the *CDH1* genomic repeat elements landscape. The rs16260 heterozygous genotype was significantly associated with a reduced risk of 1-year recurrence (*p* < 0.05). Moreover, the CG haplotype emerged as a protective factor against recurrence within two years. Illustrative immunofluorescence analysis confirmed the loss of E-cadherin and epithelial disorganization in muscle-invasive samples compared to non-invasive tumors. Notably, bioinformatic screening revealed a complex architecture of repetitive elements, including novel *Alu* insertions within Exon 16, indicating potential regulatory hubs.

**Conclusion:**

Our findings demonstrate that *CDH1* polymorphisms and haplotype structures are potential modulators of UBC recurrence. Furthermore, the identification of repetitive elements in crucial genomic segments highlights a new layer of transcriptional regulation, offering promising approaches for the prognostic stratification and molecular targeting of bladder cancer.

**Graphical abstract:**

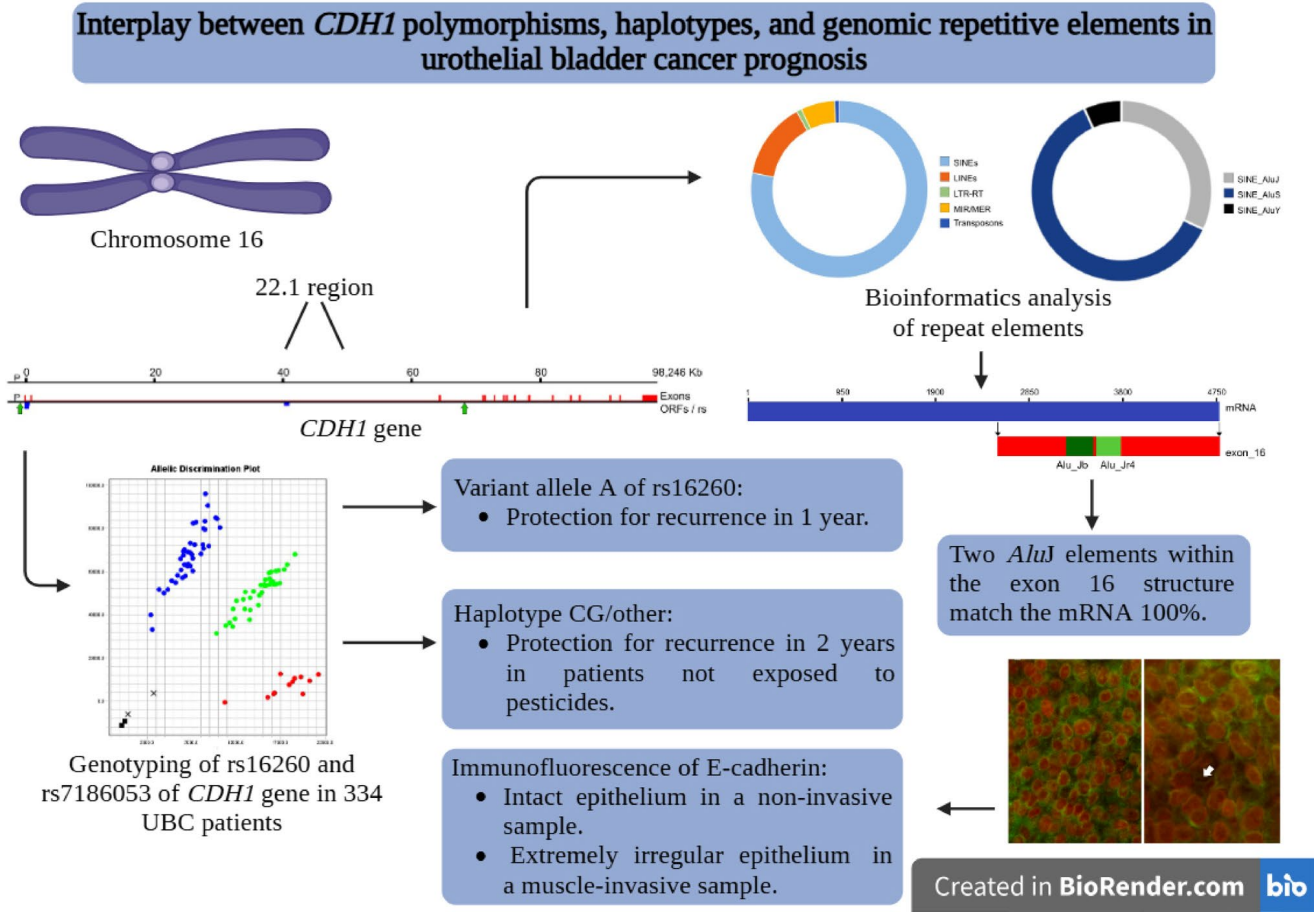

**Supplementary Information:**

The online version contains supplementary material available at 10.1007/s11033-026-12162-6.

## Introduction

Urothelial Bladder Cancer (UBC) is the ninth most common cancer worldwide [1]. According to the Global Cancer Observatory [2], the number of new cases is expected to increase 24.3% by 2030. Although this type of cancer predominates in more industrialized countries and the relative survival rate is about 79% in 5 years, the impact on patients’ daily lives is severe, and treatment is very expensive. This was the thirteenth leading cause of death in 2022, with 613,791 new cases, and approximately 200,000 deaths worldwide [1].

Advanced age, combined with exposure to carcinogens such as tobacco smoke, benzene-containing chemicals, aromatic amines, and an age-related decrease in DNA repair ability, may be the leading risk factor for UBC [3]. Smoking is a significant exposure factor, and accounts for approximately 50% of cases [4]. One of the main problems associated with UBC is the recurrence, which can occur at rates of 43% and 33% for low - and intermediate-risk non-muscle invasive bladder cancer (NMIBC), respectively, within five years. 21% of high-risk tumors have the potential to develop into muscle-invasive carcinoma (MIBC), and account for 30% of all localized bladder tumors [5]. The standard procedure for diagnosing UBC and monitoring for recurrence is cystoscopy [3], which is very uncomfortable for the patient and costly due to frequent hospitalization.

Several genetic alterations are associated with the development and progression of bladder cancer, particularly those related to cell growth factors, histone regulators, chromatin remodeling, and tumor suppression [6][7]. Among these, the E-cadherin gene *(CDH1)* plays an important role in tumor biology, encoding E-cadherin an adhesion glycoprotein with a large extracellular domain and a single transmembrane segment [8]. E-cadherin is essential for maintaining epithelial intercellular adhesion, cell polarity, and tissue architecture, thereby functioning as an important tumor suppressor [9]. Reduced E-cadherin expression compromises cell–cell adhesion and contributes to tumor progression and metastasis [9], leading to epithelial-mesenchymal transition (EMT), cancer invasion, and metastasis [10]. With EMT activation, the polygonal morphology typical of epithelial cells is lost. The cells adopt a mesenchymal morphology, and express markers associated with this state, such as neural cadherin (N-cadherin), vimentin, and fibronectin [11].

The human *CDH1* gene is approximately 100 kb in length, located on chromosome 16q22.1, and is organized into 16 exons and 15 introns [12]. Downregulation of E-cadherin in any of these regions may contribute to accelerated cell motility and invasion. Single nucleotide polymorphisms (SNPs) in the *CDH1* gene have been associated with a lack of cell adhesion and epithelial oncogenesis, as seen in bladder cancer [13]. However, this gene is extensive and contains introns rich in repetitive DNA, in particular short interspersed elements (SINEs) of the *Alu* superfamily. The presence of these DNA elements increases the possibilities to assess mechanisms of inactivation and mutations, CpG islands, disruption of transcription factors, and interference RNAs action [14].

A major challenge in the clinical management of UBC is the lack of reliable biomarkers for predicting recurrence risk. Genetic variants within the *CDH1* regulatory regions, such as the upstream SNP rs16260 (C > A) and the intronic variant rs7186053 (A > G), emerge as promising candidates for early prognostic stratification. Given that intronic and non-coding regions constitute the vast majority of the human genome, curated annotation of these segments is essential to elucidate their role in gene expression control. Consequently, identifying the genomic context of these SNPs and other cis-regulatory elements is vital for understanding *CDH1* dysregulation. This study investigates the association of *CDH1* polymorphisms and haplotype structures with UBC recurrence risk, while exploring regulatory genomic features that may modulate *CDH1* expression.

## Materials & methods

### Patient recruitment and cohort characterization

A cohort of 334 patients diagnosed with UBC via transurethral resection of bladder tumor (TURBT) was enrolled in this study. Peripheral blood samples were collected at the Cancer Hospital of Londrina (Londrina, Paraná, Brazil). Clinicopathological staging was established in accordance with the American Joint Committee on Cancer (AJCC) Tumor-Node-Metastasis (TNM) classification system, 8th edition [15]. All participants provided written informed consent and completed a standardized epidemiological questionnaire adapted from Carrano and Natarajan [16]. This study was conducted in compliance with ethical standards and received approval from the Institutional Review Board (Ethics Committee for Research on Human Subjects) of the State University of Londrina (under protocol number CAAE 47092521.2.1001.5231).

### Clinical data collection

Clinical and histopathological data were retrieved, including tumor grade based on cellular dysplasia (low-grade vs. high-grade) and muscle invasion status, categorized into muscle-invasive bladder cancer (MIBC) and non-muscle-invasive bladder cancer (NMIBC). Disease recurrence was evaluated over specific follow-up intervals: (i) within 6 months, (ii) within 1 year, and (iii) within 2 years, in addition to (iv) the occurrence of multiple recurrences. The classification of recurrent events was performed by specialized urologists at the Cancer Hospital of Londrina.

### Genomic DNA extraction

Peripheral blood was collected in EDTA (ethylenediaminetetraacetic acid) tubes, and genomic DNA was extracted using the PureLink™ Genomic DNA Mini Kit (Thermo Fisher Scientific, Waltham, MA, USA) following the manufacturer’s protocol. DNA concentration and purity were assessed via spectrophotometry using a NanoDrop™ 2000 (Thermo Fisher Scientific, Waltham, MA, USA). The 260/280 absorbance ratio was utilized to evaluate nucleic acid quality, with values between 1.8 and 2.0 indicating high purity. Subsequently, all samples were standardized to a final working concentration of 1.1 ng/µL for downstream genotyping analysis.

### *CDH1* SNP genotyping, haplotype inference, and statistical models

Two single nucleotide polymorphisms (SNPs) within the *CDH1* gene, rs16260 and rs7186053, were selected from the National Center for Biotechnology Information (NCBI) dbSNP database. Selection was based on a Minimum Allele Frequency threshold of > 0.1 in the Latin American Population 2 (LA2) cohort. Allele nomenclature, distinguishing between reference (wild-type) and variant (mutated) alleles, was established according to dbSNP [17]. For rs16260, the C allele was designated as the reference, while for rs7186053, the A allele served as the reference.

Genotyping was performed via Allelic Discrimination assays using Real-Time PCR (qPCR) on a StepOne™ system (Thermo Fisher Scientific, Waltham, MA, USA). Validated TaqMan™ hydrolysis probes (Applied Biosystems, Foster City, CA, USA) were employed: C__11934298_10 for rs16260 and C___2847279_10 for rs7186053. Each reaction mixture contained 2X Genotyping Master Mix (NZYTech, Lisbon, Portugal), 5 µL of genomic DNA (1.1 ng/µL), and the SNP-specific probe. Reagent concentrations and thermal cycling conditions were followed as previously described by da Silva et al. [18]. Haplotypes were subsequently inferred using PHASE software (version 2.1.1) [19, 20], incorporating the chromosomal coordinates of the *CDH1* variants (rs16260: chr16:68737131; rs7186053: chr16:68805390).

### Statistical analysis

The chi-square test was employed to assess whether the genotype distributions were in Hardy-Weinberg equilibrium. Multinomial logistic regression models were constructed to evaluate the association between the investigated SNPs, their haplotypes, and UBC prognosis (tumor grade, muscle invasion, and recurrence at 6, 12, and 24 months, including multiple recurrences). Furthermore, these models were adjusted to explore the interaction between genetic variants and sociodemographic or clinical factors, such as sex, smoking status [21], occupational pesticide exposure [22], and family history of cancer.

Genetic associations were evaluated using four pre-established inheritance models: (i) genotypic (reference homozygous vs. heterozygous vs. variant homozygous), to study the effect of each genotype separately on patient prognosis; (ii) dominant (reference homozygous vs. heterozygous + variant homozygous), to study the effect of carrying at least one copy of the variant allele on prognosis; (iii) recessive (reference homozygous + heterozygous vs. variant homozygous), to analyze the effect on prognosis in individuals carrying two copies of the variant allele; and (iv) overdominant (reference homozygous + variant homozygous vs. heterozygous), to see the specific effect of the heterozygous genotype on prognosis. For haplotype analysis, homozygous pairs were compared against heterozygous pairs and non-carriers, with the latter serving as the reference group. Results were reported as Odds Ratios (OR) with 95% Confidence Intervals (95% CI). Statistical significance was defined as *p* < 0.05. All statistical analyses were conducted using IBM SPSS Statistics version 26.0 (IBM Corp., Armonk, NY, USA).

### Immunofluorescent characterization of selected tumor specimens

Immunofluorescence analysis was performed on three tumor samples selected from the institutional biorepository to illustrate the interplay between tissue invasion levels and *CDH1* genetic backgrounds. The selected profiles included: (i) an NMIBC specimen harboring the rs16260-CA and rs7186053-AG genotypes (CG/AA haplotype); (ii) an MIBC specimen with the same genotypic profile (rs16260-CA, rs7186053-AG; CG/AA haplotype); and (iii) an MIBC specimen carrying homozygous variant genotypes for both SNPs (rs16260-AA and rs7186053-AA; AA/AA haplotype). These cases were chosen to demonstrate variations in E-cadherin expression across different stages of tumor progression and genotypic contexts.

Formalin-fixed, paraffin-embedded (FFPE) tissue blocks obtained via TURBT were sectioned (5mum), deparaffinized, and rehydrated. Initial hematoxylin and eosin (H&E) staining was performed to confirm the diagnosis and ensure the presence of sufficient tumor tissue. Sections underwent heat-induced antigen retrieval and were subsequently incubated overnight at 4 °C with a rat Alexa Fluor^®^ 594 anti-CD324 (E-cadherin) antibody (1:200; BioLegend, Cat: 147306). Following washes in 1× PBS, slides were mounted using 25 µL DABCO, a solution of glycerol (90%), 1,4-diaza-bicyclo (2.2.2)-octane (2.3%), 20 mm Tris–HCl, pH 8.0 (2%), 2.5 mm MgCl2 (4%) and distilled water (1.7%), and 1 µL of 2 µg mL–1 Hoescht 33,342 (Ho; Cat. No 14533, Sigma-Aldrich). Images were captured using a Leica DM4500 B microscope equipped with a DFC 300FX camera. Digital images were acquired in grayscale, pseudocolored (red for Hoechst; yellow-green for Alexa Fluor^®^ 594), and processed for contrast using GIMP (version 2.8).

### In silico characterization of repetitive elements within the *CDH1* locus

The *CDH1* genomic sequence (NC_000016.10) was retrieved from the National Center for Biotechnology Information (NCBI) database. To identify repetitive elements, including transposons, the sequence was submitted to the Censor/Giri web tool, restricted to human reference sequences. Repeats were categorized by Order, Superfamily, and Family. Exonic regions were mapped using the GCF_000001405.40 genome dataset. The precise locations of these repeats within introns or exons were determined from Censor/Giri output files and verified via multiple sequence alignment using the MUSCLE tool within the UGENE software suite (Linux).

Non-coding RNA-related sequences were identified using the RNAcentral platform, and a BED file was generated to define their coordinates. To identify putative CpG islands and open reading frames (ORFs), the *CDH1* sequence was analyzed using Cpgplot and getorf (EMBOSS platform), with results exported as BED files. AT and GC content were subsequently estimated. All genomic features — including repeats, exons, putative ORFs, non-coding sequences, and CpG islands — were visualized and compared using pyGenomeTracks [23]. Functional mRNA sequences were obtained from NCBI and aligned using MUSCLE (UGENE). Finally, the mRNA pool was screened against a custom database derived from Censor/Giri results to detect SINE insertions within translatable sequences, utilizing local BLASTn with default parameters. Figures were generated on the SRPlot platform and refined using Inkscape v. 1.4 (Linux).

## Results

### Sample description

**Fig. 1:**
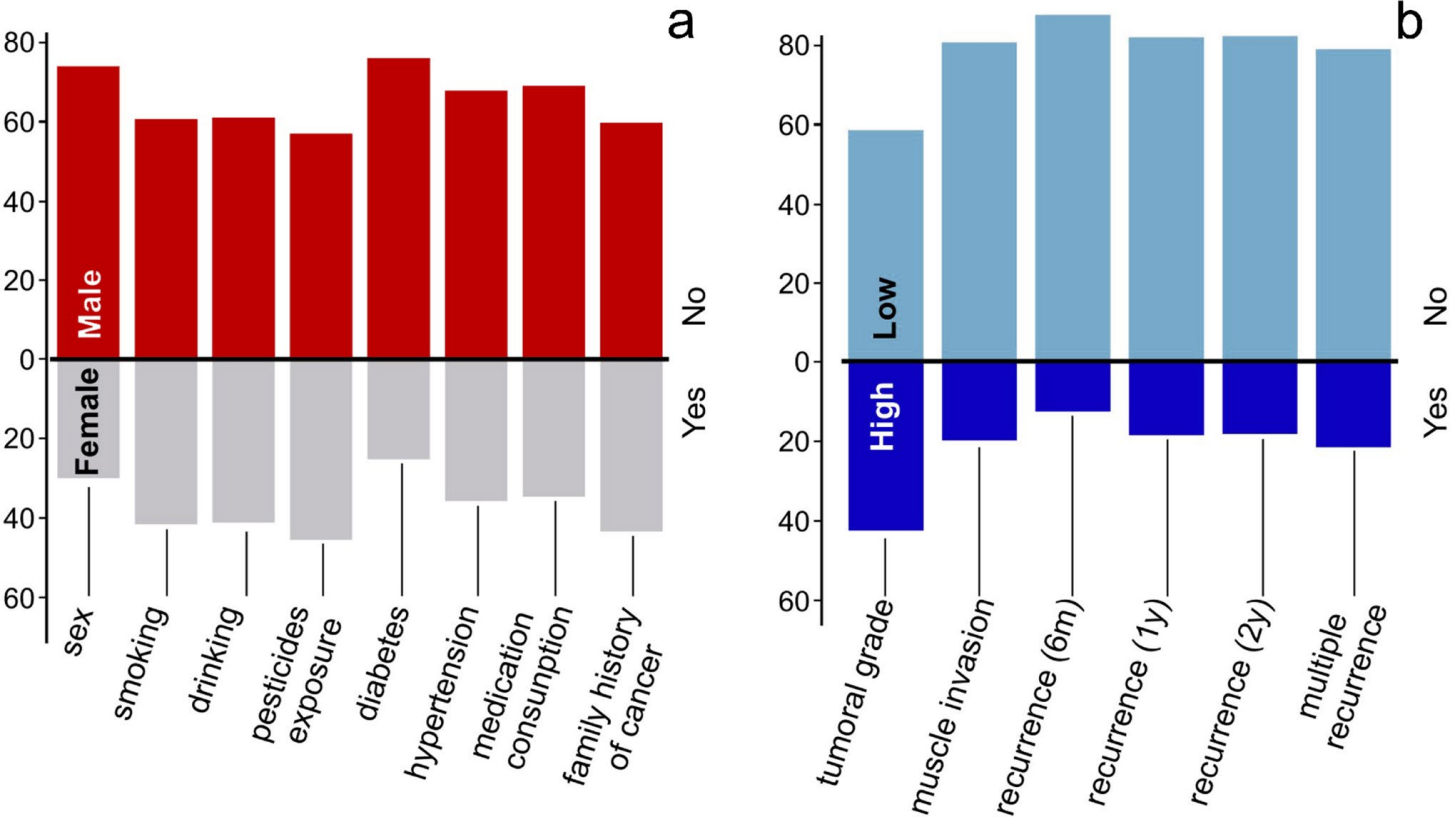
Baseline characteristics (**a**) and prognostic indicators (**b**) of the UBC sample. Note that, in most cases, the UBCs are of low severity

The study cohort comprised 334 patients, with a male predominance (*n* = 234; 70%) and a mean age of 68.61 ± 10.01 years. Regarding lifestyle and risk factors, the majority were non-smokers (60.8%) and had no prior exposure to pesticides (57.1%), although a family history of cancer was reported by 56.6% of the participants (Fig. 1a). Detailed absolute frequencies are available in Supplementary Information 1.

Clinical and prognostic analysis revealed that most malignancies were low-grade (58.6%) and classified as NMIBC (80.8%). Longitudinal follow-up showed high non-recurrence rates: 87.8% at 6 months, 82.1% at 1 year, and 82.4% at 2 years. Furthermore, 79.1% of the patients did not experience multiple recurrences (Fig. 1b; Supplementary Information 2). The relatively low 1–2 year recurrence rate of 18% observed in our study is on the lower end of the spectrum compared to broader literature, a finding primarily attributed to our limited follow-up duration.

Genotyping analysis revealed that the reference (C) and variant (A) allele frequencies for rs16260 were 75% and 25%, respectively. For rs7186053, the reference allele (A) frequency was 32%, while the variant allele (G) was 68%. Both polymorphisms were in Hardy-Weinberg equilibrium (*p* > 0.05) (χ^2^ = 3.00 for rs16260 and χ^2^ = 0.12 for rs7186056). Genotype frequencies for both SNPs across different genetic models are detailed in Supplementary Information 3.

### rs16260 showed protection against recurrence in 1 year

Logistic regression analysis of rs16260 genotypes revealed that heterozygosity (CA) was significantly associated with a protective effect against 1-year recurrence. This association was observed in both the genotypic model (*p* = 0.026; OR = 0.420; 95% CI:0.096–0.903) and the overdominant model (*p* = 0.024; OR = 0.421; 95% CI:0.199–0.893), as detailed in Table 1.

In an analysis stratified by sex, the CA genotype was found to be a protective factor against 1-year recurrence among male patients. This association was consistent across multiple genetic models: the genotypic model (*p* = 0.021; OR = 0.291; 95% CI:0.105–0.836), the dominant model (CA + AA; *p* = 0.025; OR = 0.350; 95% CI:0.140–0.876), and the overdominant model (CA; *p* = 0.026; OR = 0.313; 95% CI:0.112–0.869), as shown in Table 1.

**Table 1:** Multinomial logistic regression analysis of rs16260 and 1-year recurrence: main effects and interaction with sex in UBC patients

SNP	Parameters		Model	Genotype	OR (CI 95%)	*p-value*
rs16260	Recurrence in 1 year	No	Genotypic	CC	Reference	
		Yes		CA	0.420 (0.196–0.903)	0.026*
				AA	0.981 (0.338–2.847)	0.972
		No	Dominant	CC	Reference	
		Yes		CA + AA	0.519 (0.266–1.014)	0.055
		No	Recessive	CC + CA	Reference	
		Yes		AA	1.303 (0.458–3.706)	0.62
		No	Overdominant	CC + AA	Reference	
		Yes		CA	0.421 (0.199–0.893)	0.024*
	Recurrence in 1 year / Sex	No / Man	Genotypic	CC	Reference	
		Yes / Man		CA	0.297 (0.105–0.836)	0.021*
		Yes / Man		AA	0.633 (0.131–3.062)	0.570
		No / Man	Dominant	CC	Reference	
		Yes / Man		CA + AA	0.350 (0.140–0.876)	0.025*
		No / Man	Overdominant	CC + AA	Reference	
		Yes / Man		CA	0.313 (0.112–0.869)	0.026*

Reference: No. SNP: Single nucleotide polymorphism. OR: odds ratio. CI: confidence interval. Multinomial logistic regression. *Significance level of *p* < 0.05.

### rs7186053 did not show any association with prognostic parameters

No statistically significant associations were found between rs7186053 genotypes and tumor prognostic parameters across any of the genetic models (genotypic, dominant, recessive, and overdominant). Similarly, stratified analyses considering interactions between genotypes and sociodemographic or clinical variables yielded no significant results.

### Haplotype CG/other showed protection against recurrence in 2 years

Based on the genotyping of patients and the inference of haplotypes, we found that the CG haplotype is the most common in the population, present in 290 patients (86.8%) on at least one chromosome, followed by the AA haplotype, present in 120 patients (35.9%) on at least one chromosome. The absolute numbers are shown in Supplementary Information 4. In terms of haplotype models, the CG/CG pair is the most common occurring in 139 patients (41.6%), as shown in Supplementary Information 5.

When multinomial logistic regression was performed to assess whether the presence of the haplotype in homozygosity, heterozygosity, or its absence influenced prognostic factors, we found that the CG/other haplotype conferred protection against recurrence in 2 years in patients who were not exposed to pesticides (*p* = 0.038; OR = 0.141; 95% CI = 0.022–0.896) (Table 2).

**Table 2:** Multinomial logistic regression analysis of rs16260 and rs7186053 haplotypes with a prognostic parameter interacting with sociodemographic parameters of the UBC patients

Haplotype	Parameters		Haplotype models	OR (CI 95%)	*p*-value
CG	Recurrence in 2 years / Occupational exposure to pesticides	No / No	other/other	Reference	
		Yes / No	CG/other	0.141 (0.022–0.896)	0.038*

Reference: No. OR: odds ratio. CI: confidence interval. Multinomial logistic regression. *Significance level of *p* < 0.05

### Immunofluorescence profiles of E-cadherin protein

**Fig. 2:**
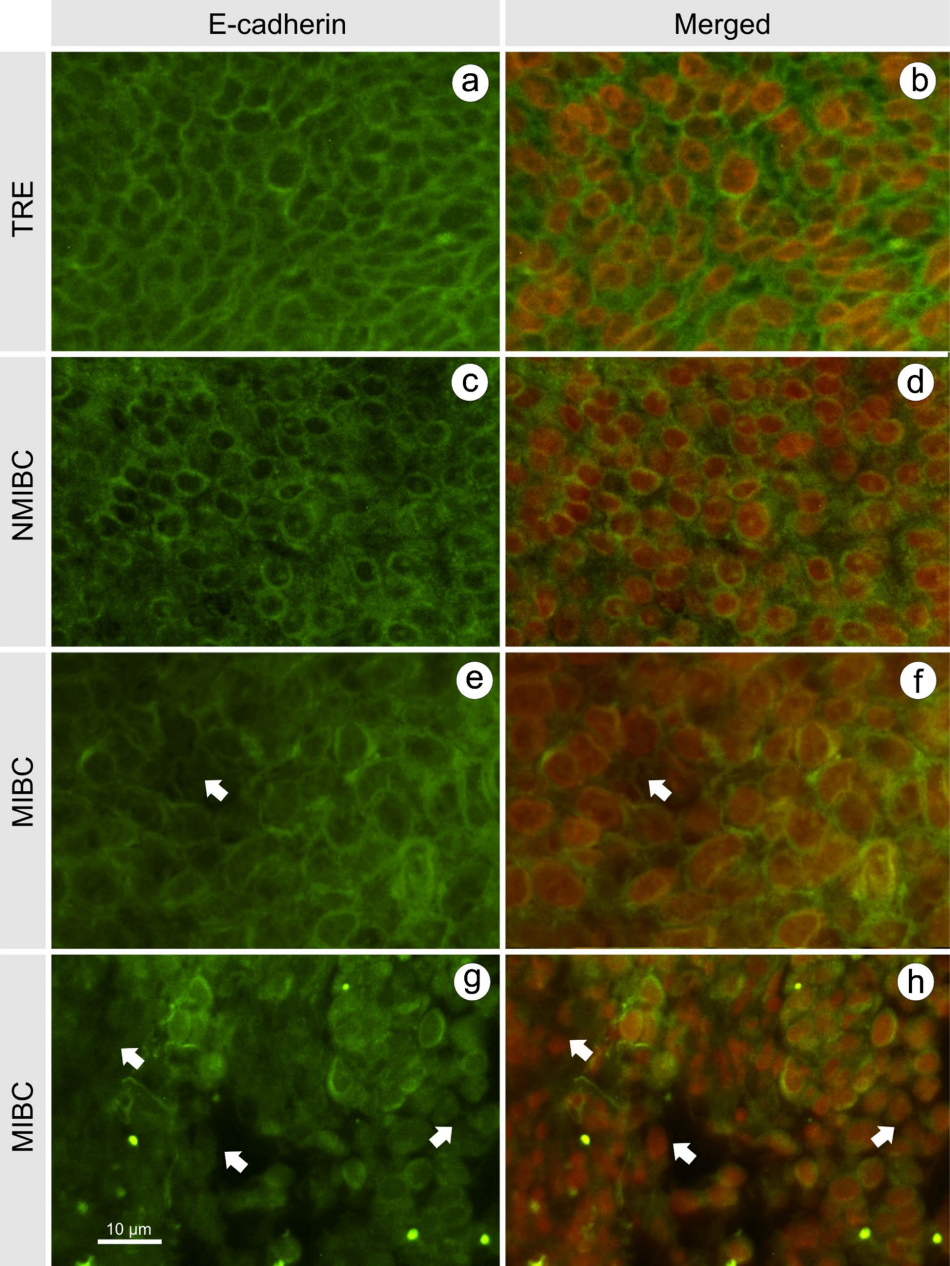
Illustrative E-cadherin immunofluorescence profiles in UBC tissue samples according to tumor stage and genotype. The first column displays E-cadherin expression (green/red), and the second column shows the merge with Ho-stained nuclei (red). (**a**–**b**) Representative profile of a tumoral regular epithelium (TRE) showing preserved protein distribution. (**c**–**d**) NMIBC sample (rs16260-CA and rs7186053-AG genotypes) exhibiting continuous E-cadherin expression surrounding epithelial cells. (**e**–**f**) MIBC sample (rs16260-CA and rs7186053-AG genotypes) showing urothelial irregularity and reduced membrane staining intensity (white arrows). (**g**–**h**) MIBC sample (rs16260-AA and rs7186053-AA genotypes) displaying advanced urothelial disorganization and focal loss of E-cadherin expression (white arrows). All images were captured at 400× magnification. TRE: tumoral regular epithelium; NMIBC: non-muscle invasive bladder cancer; MIBC: muscle invasive bladder cancer

Immunofluorescence analysis of NMIBC and MIBC samples, even if done for illustrative purposes, revealed distinct E-cadherin expression patterns across different clinical stages (Fig. 2). A uniform E-cadherin signal was observed in regular tumor epithelium (Fig. 2a, b). Regarding the relationship between tumor invasion and genotypes, patients with non-invasive disease carrying the rs16260 (CA) and rs7186053 (AG) genotypes generally exhibited uniform expression of this adhesion molecule (Fig. 2c, d). In contrast, E-cadherin expression was markedly less uniform in patients with invasive disease (Fig. 2e-h). Specifically, MIBC patients with CA (rs16260) and AG (rs7186053) genotypes displayed an irregular urothelium (Fig. 2e, f), while those with the AA (rs16260) and AA (rs7186053) genotypes showed even more pronounced urothelial disorganization (Fig. 2g, h).

### Characterization of Alu element insertions in the CDH1 locus

**Fig. 3:**
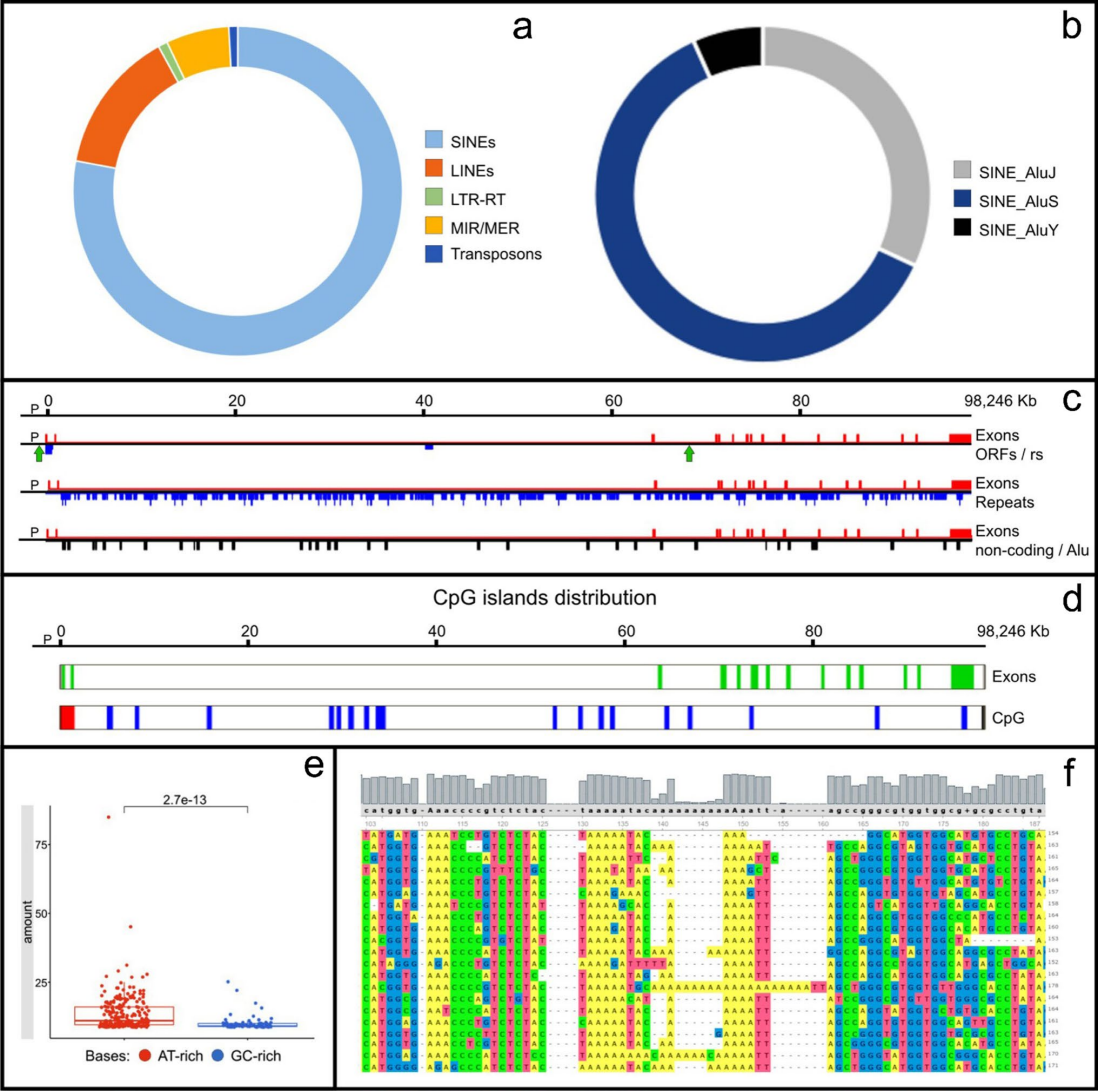
Genomic landscape of repetitive elements and nucleotide composition within the human *CDH1* gene. (**a**) Proportional distribution of repetitive elements, highlighting the predominance of SINEs. (**b**) Classification of SINE subfamilies, showing the prevalence of *Alu*S elements. (**c**) Genomic mapping of repetitive elements relative to exon positions; green arrows indicate the locations of the analyzed SNPs, and open reading frames (ORFs) are identified across the gene sequence. (**d**) Distribution of CpG islands within the *CDH1* structure; the red band denotes the region with the highest CpG density, while blue bands indicate regions with lower content. (**e**) Quantitative analysis of AT and GC dimers across gene expansion, demonstrating a high abundance of AT-rich regions. (**f**) Sequence alignment showing the localization of AT (yellow/red) and GC (blue/green) dimers within various *Alu* element sequences identified in the gene

Analysis of repetitive elements within the *CDH1* gene revealed a high prevalence of SINEs (78%), alongside trace amounts of LINEs. LTR-LTR, MIR/MER, and DNA transposons were also identified, albeit in lower proportions (7.9%; Fig. 3a). Among the SINE family, the *Alu*S subfamily predominated (61%), followed by *Alu*J and *Alu*Y (Fig. 3b). These repetitive elements were primarily located within intronic regions, with the notable exception of an *Alu* element insertion identified in Exon 16 (Fig. 3c).

Regarding CpG island distribution, a dense cluster of these dimers was identified at the 5’ end of the gene, encompassing the promoter region and the first two exons. Furthermore, additional CpG-rich regions were observed: five interspersed with exons and one localized within Exon 16 (Fig. 3d). AT-rich regions were also identified, occurring at a higher frequency than CpG islands (Fig. 3e). Comparative alignment analysis demonstrated that these AT-rich regions exhibit greater sequence variability and length than the GC-rich sites (Fig. 3f).

**Fig. 4:**
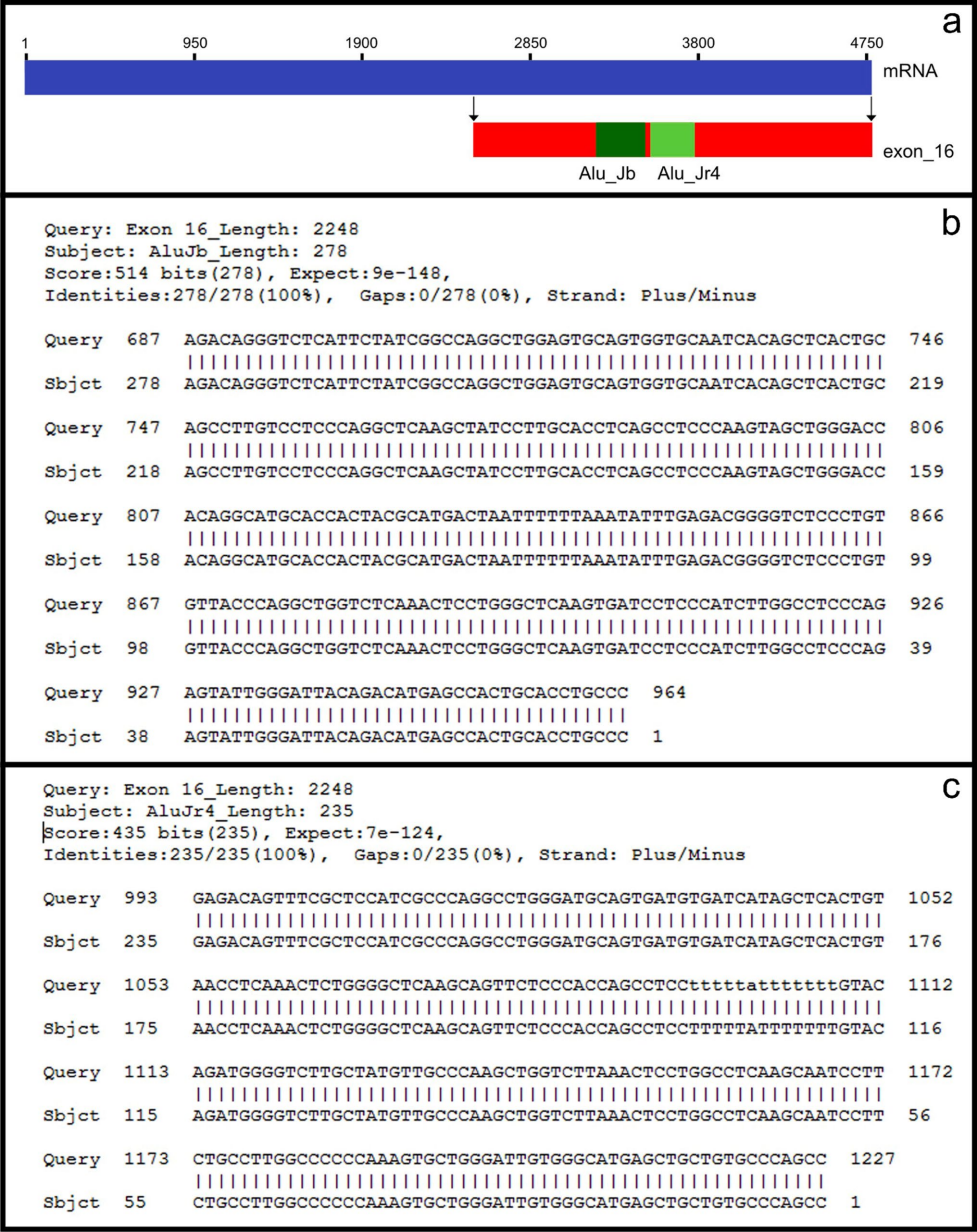
Sequence alignment between *CDH1* mRNA and Exon 16. (**a**) Mapping of the mRNA region corresponding to Exon 16, highlighting the integrated *Alu*J retrotransposons within the exonic structure. (**b**–**c**) Detailed alignment demonstrating 100% sequence identity between the two *Alu* elements and the mRNA transcript

Sequence alignment between the messenger RNA (mRNA) and CDH1 Exon 16 demonstrated that this exon accounts for nearly half of the total mRNA sequence (Fig. 4a). Furthermore, the alignment revealed that the two AluJ elements integrated into the exonic structure exhibit 100% identity with the mRNA transcript (Fig. 4b, c)

## Discussion

To our knowledge, this is the first study to evaluate *CDH1* polymorphisms (rs16260 and rs7186053) and haplotypes alongside a structural bioinformatic analysis of repetitive elements in UBC. We identified the rs16260 CA genotype and the CG haplotype as protective factors against recurrence at 1 and 2 years, respectively, with the latter showing clinical relevance in specific environmental and hereditary contexts. The observed reduction in E-cadherin-mediated adhesion in invasive tumors aligns with these genetic findings. Moreover, the identification of *Alu* elements within intronic regions and Exon 16 reveals a complex genomic architecture that may modulate *CDH1* transcriptional control, highlighting a new avenue for understanding UBC progression.

Advanced age is closely associated with prolonged exposure to carcinogens, such as tobacco smoke, occupational exposure to pesticides, aluminum, rubber, paint, and dyes, environmental exposure to X-rays, gamma rays, and arsenic, and medication and opioid use [24]. As the average age of the 234 men in our sample was 68.61 years, this reinforces the findings of Sung et al. [25], who pointed out a predominance of UBC in men over 55 years old.

In addition to environmental factors, genetic factors also play a role in the development of UBC. In this context, the polymorphism rs16260 (C > A) is located in the upstream region of the *CDH1* gene. Polymorphisms in this region are known to alter the regulation of gene transcription [26]. This is not different for rs16260, as many studies suggest that the variant allele decreases the function of the gene and increases the risk of some cancers such as cervical [27], prostate [28], gastric [29] and urothelial [30] cancers. This decrease in function is because the presence of the variant allele A alters the transcriptional activity of the gene by changing its structure, thereby reducing the access of transcription factors [31]. However, these studies are controversial. It has been shown that the risk of developing certain types of cancer, such as gastric cancer, due to the presence of the variant allele depends on the patient’s ethnicity. In this context, Caucasian patients with the variant allele had a higher risk of developing this neoplasia, but Asian patients did not [32]. Since our sample was from the northern region of the Brazilian state of Paraná, Brazil, most patients were of European and African descent, characterized as Caucasian and Black [33].

Our study found a protective association of the CA heterozygous of rs16260 for recurrences in 1 year, in both genotypic and overdominant models, in UBC patients. Recurrences occur because not all cells in the tumor are eradicated surgically or by chemotherapy, radiation, and immunotherapies to which the patient is subjected. Additionally, the inflammatory reaction that occurs after surgery can facilitate the spread of residual cancer cells. These cells adhere to the epithelium, divide again, and form a recurrent tumor mass [34]. Since previous studies suggest that the A allele may reduce *CDH1* transcription by altering transcription factor binding sites, potentially leading to decreased E-cadherin expression [31], we suggest that the reference allele C enables efficient transcription of the *CDH1* gene, thereby facilitating normal adherence of cancer cells to the rest of the urothelium, which makes their eradication more challenging. In contrast, allele A leads to reduced expression of the protein [31]. This way, the heterozygous CA has a balanced amount of E-cadherin, which can be fundamental for the recurrence occurrence.

Polymorphisms in the intronic region can alter transcription by modifying splice sites and binding sites for long non-coding RNAs (lncRNAs) [35, 36]. A polymorphism located in the third intron of the *CDH1* gene (rs7186053, A > G) appears to offer protection to some cancer types in the literature, but in our study, it did not show any role in UBC prognosis. Although this polymorphism has been associated with an increased risk of oral clefts based on haplotype and SNP analyses [37], other studies have demonstrated other actions of this mutation. The study by Jia et al. [38] indicates that patients with malignant breast neoplasms who are homozygous for the reference genotype (AA) or heterozygous (GA) for rs7186053 have favorable disease-free survival in less aggressive tumor subsets. This suggests that reference A allele may protect against the most aggressive forms of breast tumors. This polymorphism has also been analyzed in patients with endometrial and gastric cancer, but no significant influence was observed [39].

In addition, rs16260 and rs7186053 have been studied together in a haplotype model to investigate cleft palate [37, 40]. In our study, the CG haplotype was the most common, as it is the combination of the most frequently found alleles. Interestingly, the presence of the CG haplotype was associated with a protective effect for tumor recurrence in 2 years in patients without occupational exposure to pesticides. This association is consistent with our previous findings, as the C allele of rs16260 was protective for recurrence in 1 year, suggesting that genetic variants within the *CDH1* regulatory region may collectively influence disease outcomes. Haplotypes capture the combined inheritance of nearby genetic variants, and they may provide additional insight into regulatory effects that cannot be detected through single-SNP analyses alone. UBC is not a predominantly hereditary cancer, and the most significant risk factor for developing UBC is exposure to carcinogens such as pesticides [24], which showed a significant association with the CG haplotype. Therefore, the observed interaction between haplotype structure and pesticide exposure may reflect a complex interplay between genetic susceptibility and environmental factors in determining tumor recurrence risk.

Although illustrative and with no statistical validation, the presence of the adhesion protein E-cadherin was visualized in our study, proving that it is present in greater amounts in patients with a non-muscle invasive prognosis, compared to patients with invasive features. In addition, we observed that in patients with MIBC tumors, the epithelium was more disorganized and had fewer cells surrounded by the adhesion protein when they had genotypes AA for rs16260 and AA for rs7186053. This is consistent with the literature that *CDH1* gene expression decreases in patients with mutations for rs16260, which reduces the affinity of transcription factors for the gene [31].

As the *CDH1* gene is extensive, at around 100 kb and containing approximately 90% introns [12], variants and repetitive DNA are both significant factors [14]. The *Alu* superfamily of SINE-type retrotransposons was found most frequently among the repetitive elements in the gene. An *Alu* element is composed of two nearly identical arms derived from 7SL RNA monomers, which are separated by an A-rich sequence [41]. Additionally, *Alu* elements are rich in CpG dinucleotides and dysregulation of methylation levels, mainly hypomethylation, could favor non-LTR retrotransposon activation and lead to epigenetic dysfunction during oncogenesis [42]. Bioinformatic analysis revealed two large, polymorphic, AT-rich fractions in the *CDH1* gene and several CpG islands. The first of these are highly CpG-dense and have a high potential for methylation. During tumorigenesis, the promoters of many genes may undergo changes in methylation levels, which affect numerous signaling pathways [43]. However, the high GC density associated with the first two exons may be critical for silencing the *CDH1* gene and losing cell-to-cell contact, which is associated with UBC aggressiveness.

*Alu* elements can be retrotransposed into or close to any coding gene sequence [44]. This process may be a source of diversity, as it can affect splicing [45]. *Alu* elements favor this process, particularly when they are in an antisense orientation, as the 5’ and 3’ splicing signals are then more likely to be recognized [46]. Bioinformatic data revealed several *Alu* elements in intronic regions and two inserted in Exon 16 of the *CDH1* gene. Non-LTR retrotransposon insertions into different regions of the genome can interfere with gene expression through various pathways, ranging from regions lacking repair due to endo-exonuclease activity to major deletions and rearrangements. However, retrotransposon insertions into coding regions, such as *Alu* sequences into the E-cadherin gene as observed here, can cause frameshifts in exonic regions of mRNA, which can affect gene expression. Similarly, the insertion of *Alu* elements into intronic regions could create additional splice sites and consequently interfere with transcriptional integrity [42]. These findings suggest that exonization may drive the incorporation of these elements into the coding sequence. Collectively, our observations underscore the necessity of evaluating cancer-related genes beyond single-nucleotide variants, considering the broader genomic architecture. Future research integrating functional assays and transcriptomic profiling will be essential to provide mechanistic insights into the regulatory roles of these repetitive elements in *CDH1* modulation and their subsequent impact on UBC progression.

While the number of SNPs analyzed restricted the scope of the haplotype analysis, this limitation was mitigated by the robust sample size, the stringency of the statistical methods, and the high quality of the clinical data available. The relatively low recurrence rate observed in our study represents a limitation and is primarily a reflection of our short follow-up duration. Furthermore, the integration of a pioneering bioinformatic approach provided a uniquely comprehensive characterization of the *CDH1* gene, significantly adding to the study’s scientific value.

## Conclusion

In conclusion, our study demonstrates that rs16260 and the CG haplotype are significantly associated with a reduced risk of early recurrence in UBC patients, particularly within specific clinical subgroups. These genetic associations, coupled with the identification of a high density of *Alu* retrotransposons in key areas like Exon 16, suggest that the genomic architecture of *CDH1* plays a critical role in modulating tumor behavior. Although the functional mechanisms of these repetitive elements require further elucidation, our findings highlight them as promising, underexplored targets for biomarker discovery and personalized prognostic strategies in bladder cancer.

## Supplementary Information

Below is the link to the electronic supplementary material.

Supplementary Material 1

Supplementary Material 2

Supplementary Material 3

Supplementary Material 4

Supplementary Material 5

## Data Availability

No datasets were generated or analysed during the current study.

## Abbreviations

CDH1: E-cadherin gene
CI: Confidence interval
dbSNP: Single Nucleotide Polymorphism Database
DNA: deoxyribonucleic acid
EDTA: Ethylenediaminetetraacetic acid
EMT: Epithelial-mesenchymal transition
Ho: Hoescht
LA2: Latin American population 2
LINEs: Long interspersed nuclear elements
MIBC: Muscle-invasive carcinoma
N: Total number of patients
N-cadherin: Neural cadherin
NMIBC: Non-muscle invasive bladder cancer
OR: Odds ratio
ORFs: Open reading frames
PCR: Polymerase chain reaction
RNAm: Messenger RNA
SINEs: Short interspersed elements
SNP: Single nucleotide polymorphism
TNM: Tumor-node-metastasis
TURB: Transurethral bladder resection
UBC: Urothelial bladder cancer
