# Supplementary material for: Interplay between CDH1 polymorphisms, haplotypes, and genomic repetitive elements in urothelial bladder cancer prognosis
Source: Mol Biol Rep. 2026 Jun 23;53(1):990. doi: 10.1007/s11033-026-12162-6 (PMC13290822; doi:10.1007/s11033-026-12162-6)
Supplement: Supplementary file 1 — Supplementary Material 1 [file 11033_2026_12162_MOESM1_ESM.docx]

**Interplay between *CDH1* polymorphisms, haplotypes, and genomic repetitive elements in urothelial bladder cancer prognosis**

Laís Capelasso Lucas Pinheiro^1^, Maria Alice Feitosa de Souza Martins^1^, Maria Fernanda Vicente Turim^1^, Isabely Mayara da Silva^1^, Janaina Nicolau de Oliveira^2^, Fernando Terziotti^3^, Juliana Mara Serpeloni^1^, Karen Brajão de Oliveira^2^, André Luís Laforga Vanzela^4^ and Roberta Losi Guembarovski^1^*.

^1^ Laboratory of Mutagenesis and Oncogenetics, Department of General Biology, Londrina State University, Londrina, PR, Brazil

^2^ Laboratory of Molecular Genetics and Immunology, Department of Pathological Sciences, Londrina State University, Londrina, PR, Brazil

^3^ Cancer Hospital of Londrina – HCL, Londrina, PR, Brazil

^4^ Laboratory of Cytogenetics and Plant Diversity, Department of General Biology, Londrina State University, Londrina, PR, Brazil

*Corresponding author: Tel: +55 (43) 33715149; E-mail: robertalosi@uel.br; Address: Celso Garcia Cid Highway, PR-445, Km 380 - University Campus, Londrina - PR, Brazil (zip code: 86057-970).

**Supplementary Material 1.** Table with the sociodemographic and medical characteristics of patients with UBC

| **Sociodemographic and Medical Characteristics** | | **Patients** | |
| --- | --- | --- | --- |
|  |  | **N** | **(%)** |
| Sex | Woman | 100 | 29.9 |
|  | Man | 234 | 70.1 |
|  | Total | **334** | 100 |
| Smoking Habit | No | 203 | 60.8 |
|  | Yes | 131 | 39.2 |
|  | Total | **334** | 100 |
| Drinking Habit | No | 71 | 61.2 |
|  | Yes | 45 | 38.8 |
|  | Total | 116 | 100 |
| Occupational Exposure to Pesticides | No | 109 | 57.1 |
|  | Yes | 82 | 42.9 |
|  | Total | 191 | 100 |
| Diabetes | No | 148 | 76.3 |
|  | Yes | 46 | 23.7 |
|  | Total | 194 | 100 |
| Hypertension | No | 71 | 35.7 |
|  | Yes | 128 | 64.3 |
|  | Total | 199 | 100 |
| Daily Medication Consumption | No | 80 | 34.6 |
|  | Yes | 151 | 65.4 |
|  | Total | 231 | 100 |
| Family History of Cancer | No | 106 | 43.4 |
|  | Yes | 135 | 56.6 |
|  | Total | 241 | 100 |

N: Number of patients. The parameters of all 334 patients could not be evaluated due to the lack of information in medical records and personal questionnaires. The total number of patients is bold.
