# Supplementary material for: Interplay between CDH1 polymorphisms, haplotypes, and genomic repetitive elements in urothelial bladder cancer prognosis
Source: Mol Biol Rep. 2026 Jun 23;53(1):990. doi: 10.1007/s11033-026-12162-6 (PMC13290822; doi:10.1007/s11033-026-12162-6)
Supplement: Supplementary file 2 — Supplementary Material 2 [file 11033_2026_12162_MOESM2_ESM.docx]

**Interplay between *CDH1* polymorphisms, haplotypes, and genomic repetitive elements in urothelial bladder cancer prognosis**

Laís Capelasso Lucas Pinheiro^1^, Maria Alice Feitosa de Souza Martins^1^, Maria Fernanda Vicente Turim^1^, Isabely Mayara da Silva^1^, Janaina Nicolau de Oliveira^2^, Fernando Terziotti^3^, Juliana Mara Serpeloni^1^, Karen Brajão de Oliveira^2^, André Luís Laforga Vanzela^4^ and Roberta Losi Guembarovski^1^*.

^1^ Laboratory of Mutagenesis and Oncogenetics, Department of General Biology, Londrina State University, Londrina, PR, Brazil

^2^ Laboratory of Molecular Genetics and Immunology, Department of Pathological Sciences, Londrina State University, Londrina, PR, Brazil

^3^ Cancer Hospital of Londrina – HCL, Londrina, PR, Brazil

^4^ Laboratory of Cytogenetics and Plant Diversity, Department of General Biology, Londrina State University, Londrina, PR, Brazil

*Corresponding author: Tel: +55 (43) 33715149; E-mail: robertalosi@uel.br; Address: Celso Garcia Cid Highway, PR-445, Km 380 - University Campus, Londrina - PR, Brazil (zip code: 86057-970).

**Supplementary Material 4.** Table with the distribution of haplotype pairs in the UBC samples.

| **Gene** | **SNPs** | **Haplotype** | **Haplotype Model** | **Patients** | |
| --- | --- | --- | --- | --- | --- |
|  |  |  |  | **N** | **(%)** |
| *CDH1* | rs16260 and rs7186053 | CG | CG/CG | 139 | 41.6 |
|  |  |  | CG/other (CA, AG or AA) | 151 | 45.2 |
|  |  |  | other/other | 44 | 13.2 |
|  |  | CA | CA/CA | 6 | 1.8 |
|  |  |  | CA/other (CG, AG or AA) | 63 | 18.9 |
|  |  |  | other/other | 265 | 79.3 |
|  |  | AG | AG/AG | 5 | 1.5 |
|  |  |  | AG/other (CG, CA or AA) | 16 | 4.8 |
|  |  |  | other/other | 313 | 93.7 |
|  |  | AA | AA/AA | 18 | 5.4 |
|  |  |  | AA/other (CG, CA or AG) | 102 | 30.5 |
|  |  |  | other/other | 214 | 64.1 |

N: Number of patients. SNP: single nucleotide polymorphism.
