# Supplementary material for: Interplay between CDH1 polymorphisms, haplotypes, and genomic repetitive elements in urothelial bladder cancer prognosis
Source: Mol Biol Rep. 2026 Jun 23;53(1):990. doi: 10.1007/s11033-026-12162-6 (PMC13290822; doi:10.1007/s11033-026-12162-6)
Supplement: Supplementary file 3 — Supplementary Material 3 [file 11033_2026_12162_MOESM3_ESM.docx]

**Interplay between *CDH1* polymorphisms, haplotypes, and genomic repetitive elements in urothelial bladder cancer prognosis**

Laís Capelasso Lucas Pinheiro^1^, Maria Alice Feitosa de Souza Martins^1^, Maria Fernanda Vicente Turim^1^, Isabely Mayara da Silva^1^, Janaina Nicolau de Oliveira^2^, Fernando Terziotti^3^, Juliana Mara Serpeloni^1^, Karen Brajão de Oliveira^2^, André Luís Laforga Vanzela^4^ and Roberta Losi Guembarovski^1^*.

^1^ Laboratory of Mutagenesis and Oncogenetics, Department of General Biology, Londrina State University, Londrina, PR, Brazil

^2^ Laboratory of Molecular Genetics and Immunology, Department of Pathological Sciences, Londrina State University, Londrina, PR, Brazil

^3^ Cancer Hospital of Londrina – HCL, Londrina, PR, Brazil

^4^ Laboratory of Cytogenetics and Plant Diversity, Department of General Biology, Londrina State University, Londrina, PR, Brazil

*Corresponding author: Tel: +55 (43) 33715149; E-mail: robertalosi@uel.br; Address: Celso Garcia Cid Highway, PR-445, Km 380 - University Campus, Londrina - PR, Brazil (zip code: 86057-970).

**Supplementary Material 2.** Table with the prognostic parameters of patients with UBC.

| **Prognostic Parameters** | | **Patients** | |
| --- | --- | --- | --- |
|  |  | **N** | **(%)** |
| Tumor Grade | Low | 157 | 58.6 |
|  | High | 111 | 41.4 |
|  | Total | 268 | 100 |
| Muscle Invasion | No | 189 | 80.8 |
|  | Yes | 32 | 19.2 |
|  | Total | 221 | 100 |
| Recurrence in 6 Months | No | 230 | 87.8 |
|  | Yes | 32 | 12.2 |
|  | Total | 262 | 100 |
| Recurrence in 1 Year | No | 215 | 82.1 |
|  | Yes | 47 | 17.9 |
|  | Total | 262 | 100 |
| Recurrence in 2 Years | No | 216 | 82.4 |
|  | Yes | 46 | 17.6 |
|  | Total | 262 | 100 |
| Multiple Recurrence | No | 208 | 79.1 |
|  | Yes | 55 | 20.9 |
|  | Total | 263 | 100 |

N: Number of patients. The parameters of all 334 patients could not be evaluated due to the lack of information in medical records and personal questionnaires.
