# Supplementary material for: Interplay between CDH1 polymorphisms, haplotypes, and genomic repetitive elements in urothelial bladder cancer prognosis
Source: Mol Biol Rep. 2026 Jun 23;53(1):990. doi: 10.1007/s11033-026-12162-6 (PMC13290822; doi:10.1007/s11033-026-12162-6)
Supplement: Supplementary file 4 — Supplementary Material 4 [file 11033_2026_12162_MOESM4_ESM.docx]

**Interplay between *CDH1* polymorphisms, haplotypes, and genomic repetitive elements in urothelial bladder cancer prognosis**

Laís Capelasso Lucas Pinheiro^1^, Maria Alice Feitosa de Souza Martins^1^, Maria Fernanda Vicente Turim^1^, Isabely Mayara da Silva^1^, Janaina Nicolau de Oliveira^2^, Fernando Terziotti^3^, Juliana Mara Serpeloni^1^, Karen Brajão de Oliveira^2^, André Luís Laforga Vanzela^4^ and Roberta Losi Guembarovski^1^*.

^1^ Laboratory of Mutagenesis and Oncogenetics, Department of General Biology, Londrina State University, Londrina, PR, Brazil

^2^ Laboratory of Molecular Genetics and Immunology, Department of Pathological Sciences, Londrina State University, Londrina, PR, Brazil

^3^ Cancer Hospital of Londrina – HCL, Londrina, PR, Brazil

^4^ Laboratory of Cytogenetics and Plant Diversity, Department of General Biology, Londrina State University, Londrina, PR, Brazil

*Corresponding author: Tel: +55 (43) 33715149; E-mail: robertalosi@uel.br; Address: Celso Garcia Cid Highway, PR-445, Km 380 - University Campus, Londrina - PR, Brazil (zip code: 86057-970).

**Supplementary Material 3.** Frequency of SNPs rs16260 and rs7186053 in genetic models in urothelial bladder cancer patients.

| **SNP** | **Model** | **Genotype** | **Patients** | | **Frequency (%)** | |
| --- | --- | --- | --- | --- | --- | --- |
|  |  |  |  | |  | |
|  |  |  | **N** | **(%)** | **Global** | **LA2** |
| rs16260  (C>A) | Genotypic | CC | 196 | 58.7 |  |  |
|  |  | CA | 112 | 33.5 |  |  |
|  |  | AA | 26 | 7.8 |  |  |
|  |  | C | 504 | 75.0 | 72.5 | 71.6 |
|  |  | A | 164 | 25.0 | 27.5 | 28.4 |
|  | Dominant | CC | 196 | 58.7 |  |  |
|  |  | CA + AA | 138 | 41.3 |  |  |
|  | Recessive | CC + CA | 308 | 92.2 |  |  |
|  |  | AA | 26 | 7.8 |  |  |
|  | Overdominant | CC + AA | 222 | 66.5 |  |  |
|  |  | CA | 112 | 33.5 |  |  |
| rs7186053  (A>G) | Genotypic | AA | 35 | 10.5 |  |  |
|  |  | AG | 142 | 42.5 |  |  |
|  |  | GG | 157 | 47.0 |  |  |
|  |  | A | 212 | 32.0 | 40.9 | 41.7 |
|  |  | G | 456 | 68.0 | 59.1 | 58.3 |
|  | Dominant | AA | 35 | 10.5 |  |  |
|  |  | AG + GG | 299 | 89.5 |  |  |
|  | Recessive | AA + AG | 177 | 53.0 |  |  |
|  |  | GG | 157 | 47.0 |  |  |
|  | Overdominant | AA + GG | 192 | 57.5 |  |  |
|  |  | AG | 142 | 42.5 |  |  |

SNP: Single nucleotide polymorphism. N: total number of patients. LA2: Latin American population 2.
