# Supplementary material for: Interplay between CDH1 polymorphisms, haplotypes, and genomic repetitive elements in urothelial bladder cancer prognosis
Source: Mol Biol Rep. 2026 Jun 23;53(1):990. doi: 10.1007/s11033-026-12162-6 (PMC13290822; doi:10.1007/s11033-026-12162-6)

**Interplay between *CDH1* polymorphisms, haplotypes, and genomic repetitive elements in urothelial bladder cancer prognosis**

Laís Capelasso Lucas Pinheiro^1^, Maria Alice Feitosa de Souza Martins^1^, Maria Fernanda Vicente Turim^1^, Isabely Mayara da Silva^1^, Janaina Nicolau de Oliveira^2^, Fernando Terziotti^3^, Juliana Mara Serpeloni^1^, Karen Brajão de Oliveira^2^, André Luís Laforga Vanzela^4^ and Roberta Losi Guembarovski^1^*.

^1^ Laboratory of Mutagenesis and Oncogenetics, Department of General Biology, Londrina State University, Londrina, PR, Brazil

^2^ Laboratory of Molecular Genetics and Immunology, Department of Pathological Sciences, Londrina State University, Londrina, PR, Brazil

^3^ Cancer Hospital of Londrina – HCL, Londrina, PR, Brazil

^4^ Laboratory of Cytogenetics and Plant Diversity, Department of General Biology, Londrina State University, Londrina, PR, Brazil

*Corresponding author: Tel: +55 (43) 33715149; E-mail: robertalosi@uel.br; Address: Celso Garcia Cid Highway, PR-445, Km 380 - University Campus, Londrina - PR, Brazil (zip code: 86057-970).

**Supplementary Material 5.** Distribution of haplotype pairs in the UBC samples. The percentage of haplotype models in the samples shows that the more distant the pair, the more frequently it appears in the population. The set of less frequent haplotypes, indicated as 'others', appears at a high frequency, which would not be the case if they were estimated individually.


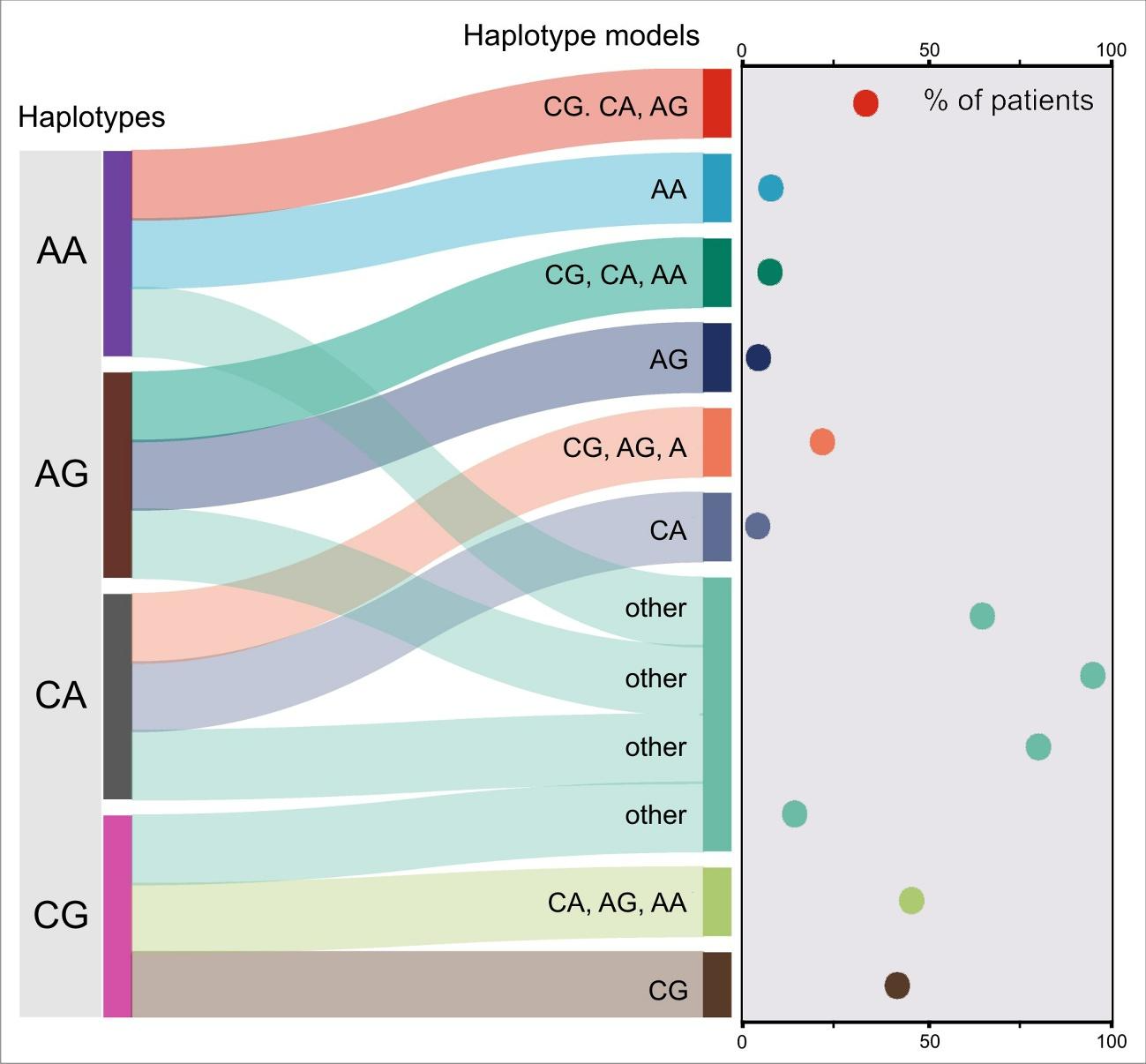

Supplement: Supplementary file 5 — Supplementary Material 5 [file 11033_2026_12162_MOESM5_ESM.docx]
